# Intersection of coral molecular responses to a localized mortality event and ex situ deoxygenation

**DOI:** 10.1002/ece3.11275

**Published:** 2024-04-23

**Authors:** Marie E. Strader, Rachel M. Wright, Ariel K. Pezner, Marissa F. Nuttall, Hannah E. Aichelman, Sarah W. Davies

**Affiliations:** ^1^ Department of Biology Texas A&M University College Station Texas USA; ^2^ Department of Biological Sciences Southern Methodist University Dallas Texas USA; ^3^ Smithsonian Marine Station Fort Pierce Florida USA; ^4^ Flower Garden Banks National Marine Sanctuary Galveston Texas USA; ^5^ Department of Biology Boston University Boston Massachusetts USA

**Keywords:** coral, deoxygenation, Flower Garden Banks, gene expression, localized mortality event, *Orbicella* spp.

## Abstract

In July 2016, East Bank of Flower Garden Banks (FGB) National Marine Sanctuary experienced a localized mortality event (LME) of multiple invertebrate species that ultimately led to reductions in coral cover. Abiotic data taken directly after the event suggested that acute deoxygenation contributed to the mortality. Despite the large impact of this event on the coral community, there was no direct evidence that this LME was driven by acute deoxygenation, and thus we explored whether gene expression responses of corals to the LME would indicate what abiotic factors may have contributed to the LME. Gene expression of affected and unaffected corals sampled during the mortality event revealed evidence of the physiological consequences of the LME on coral hosts and their algal symbionts from two congeneric species (*Orbicella franksi* and *Orbicella faveolata*). Affected colonies of both species differentially regulated genes involved in mitochondrial regulation and oxidative stress. To further test the hypothesis that deoxygenation led to the LME, we measured coral host and algal symbiont gene expression in response to ex situ experimental deoxygenation (control = 6.9 ± 0.08 mg L^−1^, anoxic = 0.083 ± 0.017 mg L^−1^) in healthy *O. faveolata* colonies from the FGB. However, this deoxygenation experiment revealed divergent gene expression patterns compared to the corals sampled during the LME and was more similar to a generalized coral environmental stress response. It is therefore likely that while the LME was connected to low oxygen, it was a series of interconnected stressors that elicited the unique gene expression responses observed here. These in situ and ex situ data highlight how field responses to stressors are unique from those in controlled laboratory conditions, and that the complexities of deoxygenation events in the field likely arise from interactions between multiple environmental factors simultaneously.

## Graphical abstract

In July 2016, East Bank of Flower Garden Banks (FGB) National Marine Sanctuary experienced a localized mortality event (LME) of multiple invertebrate species that ultimately led to reductions in coral cover, the source of which was unknown but suspected to be related to deoxygenation. Gene expression of reef‐building corals directly following the event was measured, as well as in a controlled deoxygenation experiment ex situ. We found that gene expression patterns during the LME and were more similar to a generalized coral environmental stress response than to deoxygenation, therefore, while the LME was connected to low oxygen, it was more likely due to a series of interconnected stressors that elicited the unique gene expression responses observed here.

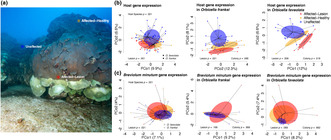

## 1 BACKGROUND

While ocean warming continues to be the dominant threat to marine systems (Intergovernmental Panel on Climate Change [IPCC], 2022), warming can interact with local stressors to create additional indirect axes of stress (França et al., 2020). For example, ocean warming and localized nutrient input in coastal ecosystems can fuel algal production of dissolved organic matter (DOM), leading to bloom‐associated microbes that can create acute deoxygenation events (Nelson & Altieri, 2019). Locally, warming also leads to decreased oxygen solubility, increased stratification, and increased respiration rates, all of which reduce the amount of available dissolved oxygen in the water column (Altieri & Gedan, 2015; Bopp et al., 2013; Breitburg et al., 2018; Keeling et al., 2010). In a positive feedback loop, high temperatures can exacerbate the effects of physiological hypoxia in marine macrofauna, as higher temperatures increase metabolic rates and thus oxygen demand (Deutsch et al., 2015; Pörtner et al., 2017) and can also decrease low oxygen tolerance (Vaquer‐Sunyer & Duarte, 2011). Thus, warming and deoxygenation are tightly coupled in marine environments, increasing the magnitude of low oxygen‐related stress in organisms experiencing deoxygenation events (Altieri & Gedan, 2015; Deutsch et al., 2015; Nelson & Altieri, 2019; Pörtner et al., 2017; Vaquer‐Sunyer & Duarte, 2011). Because of these interactive effects, mass mortality events due to low oxygen stress are commonly observed during periods of extended warming (Andréfouët et al., 2015) and dead zones are more commonly found in regions experiencing higher rates of warming (Altieri & Gedan, 2015). In addition to the complex interactions between warming, nutrients, and oxygen, coral reef geology and hydrodynamics also contribute to the risk of deoxygenation events through modulation of water residence time and flow through reef systems (Falter et al., 2013; Lowe & Falter, 2015). Thus, reefs that are particularly shallow, near sources of freshwater runoff, or are closed‐off from open ocean flow may be at higher risk of experiencing low oxygen conditions. While most dead zones and deoxygenation events have historically been characterized in temperate systems (Altieri et al., 2017), it has been shown that they are also pervasive in the tropics and sub‐tropics, with one study reporting up to 84% of coral reefs surveyed having experienced weak to moderate low oxygen and up to 94% of these reefs expected to experience moderate to severe hypoxia by the end of the century (Pezner et al., 2023). Because long‐term monitoring efforts often do not include oxygen (but see Pezner et al., 2023), most deoxygenation events go unnoticed until mass mortality is observed. Therefore, the contributions of deoxygenation events to marine ecosystem health are likely underestimated and remain understudied (Nelson & Altieri, 2019; Pezner et al., 2023).

Mass mortality on coral reefs attributed to low oxygen events has been observed across the globe (Nelson & Altieri, 2019), including the Caribbean (Altieri et al., 2017; Johnson, Swaminathan, et al., 2021; Kealoha et al., 2020), Eastern Pacific (Guzmán et al., 1990), and Indo‐Pacific (Andréfouët et al., 2015; Raj et al., 2020). Community‐level responses to acute deoxygenation events often include mass dispersal of fish and mega‐fauna as well as movement of motile invertebrates to the surface, while benthic sessile organisms with few mechanisms of rapid dispersal face mass mortality (Altieri et al., 2017; Johnson et al., 2018; Nelson & Altieri, 2019; Vaquer‐Sunyer & Duarte, 2011). On some reefs, such as those in Bocas del Toro, Panamá, deoxygenation events occur seasonally as a result of stratification in the bay (Adelson et al., 2022; Altieri et al., 2017; Johnson et al., 2018; Johnson, Swaminathan, et al., 2021). Though variable in their severity and duration, the most severe deoxygenation events in Bocas del Toro have led to mass mortality of reef organisms (Altieri et al., 2017; Johnson et al., 2018; Johnson, Swaminathan, et al., 2021). For example, in 2010, a severe deoxygenation event led to the mortality of most corals below 10–12 m depth (Altieri et al., 2017). Following a similar event in 2017, the shallow reef communities of Bocas del Toro were found to be more resilient to low oxygen environments compared to the adjacent deeper reef coral community, although community composition and species‐specific responses to deoxygenation varied substantially between these two reef habitats (Johnson, Scott, et al., 2021). While microbial communities showcased dramatic recovery to baseline communities within 1 month of the deoxygenation event, coral community structure faced long‐term shifts and appeared less resilient even after 1 year of recovery (Johnson, Scott, et al., 2021). Severe deoxygenation events such as these are likely to drive coral community shifts as ocean temperatures increase and these localized events become more frequent and severe globally (Hughes et al., 2010; Pezner et al., 2023).

From the limited field data available, reef‐building corals exposed to low oxygen in situ commonly exhibit tissue loss and classical symptoms of the coral stress response, including reductions in symbiont densities (bleaching), maximum quantum yield, and chlorophyll concentrations (Johnson, Scott, et al., 2021; Raj et al., 2020). More diverse coral physiological responses have been characterized in laboratory studies of experimental low oxygen exposure, ranging from changes in gene expression and photosynthetic rates to DNA damage and reductions in calcification (e.g., Alderdice et al., 2021; Deleja et al., 2022; Wijgerde et al., 2012, 2014; for reviews, see Nelson & Altieri, 2019; Pezner et al., 2023). Even short‐term exposure to decreased oxygen concentrations (<12 h) can lead to significant physiological consequences in terms of oxygen consumption (Gravinese et al., 2022) and calcification rates (Wijgerde et al., 2012, 2014). Additionally, laboratory studies have revealed that corals may cope with exposure to low oxygen concentrations by switching from aerobic to anaerobic metabolic pathways (Murphy & Richmond, 2016; Nelson & Altieri, 2019; Wooldridge, 2013). For example, *Montipora capitata* corals exposed to anoxic (0 mg O_2_ L^−1^) conditions for 12 h increased their expression of alanopine dehydrogenase and strombine dehydrogenase, both of which are enzymes involved in anaerobic respiration (Murphy & Richmond, 2016). While there are a handful of physiological studies characterizing coral responses to low oxygen, understanding the molecular responses of corals to low oxygen exposure could further contribute to the creation of intervention strategies for both deoxygenation and other lesion‐forming threats to coral colonies, such as disease outbreaks. For example, *Acropora tenius* exposed to deoxygenation‐reoxygenation stress over a day‐night cycle was found to possess a hypoxia‐inducible factor (HIF)‐mediated hypoxia response system (HRS) homologous to other metazoans, suggesting that this suite of genes may include markers for deoxygenation stress (Alderdice et al., 2021).

While controlled tank experiments have revealed specific physiological and molecular responses of corals to deoxygenation (e.g., Alderdice et al., 2021; Gravinese et al., 2022; Johnson, Swaminathan, et al., 2021; Wijgerde et al., 2012, 2014), they are limited in their ability to fully simulate the oxygen dynamics experienced during deoxygenation events in situ on reefs. Conventionally, ‘hypoxia’ is defined as oxygen levels less than 2.0 mg O_2_ L^−1^; however, studies in temperate organisms have shown that tolerance to low oxygen varies significantly between and within taxonomic groups (Vaquer‐Sunyer & Duarte, 2011). Furthermore, experimental studies of tropical corals have revealed variable tolerances to low oxygen between coral species. For example, *Acropora* corals experienced lesions and partial tissue loss under oxygen as high as 4 mg O_2_ L^−1^ (Haas et al., 2014) whereas *Orbicella* corals were unaffected by oxygen concentrations as low as 0.5 mg O_2_ L^−1^ (Johnson, Swaminathan, et al., 2021). In addition, natural temporal and spatial variation in oxygen can regularly expose organisms in the field to ‘hypoxic’ conditions (Camp et al., 2017; Nelson & Altieri, 2019; Pezner et al., 2023; Wallace et al., 2021), likely leading to taxa‐ and reef‐specific thresholds to low oxygen exposure (Hughes et al., 2020). For example, seagrass meadows, mangroves, and salt marsh habitats regularly experience diel acidic and hypoxic conditions (Camp et al., 2017; Giomi et al., 2023; Pezner et al., 2023; Wallace et al., 2021). While the duration of deoxygenation events is not well documented for tropical marine systems, they are estimated to last for days to weeks in temperate systems (Diaz & Rosenberg, 2008). Biotic factors can also influence the magnitude of oxygen available to corals, as algal overgrowth can lead to nighttime low oxygen exposure (Murphy & Richmond, 2016). Therefore, while corals can survive extremely low oxygen conditions for hours to 1 week in controlled lab experiments depending on the species (Dodds et al., 2007; Haas et al., 2014; Johnson, Swaminathan, et al., 2021; Mayer, 1917; Weber et al., 2012), such experiments are unable to account for the complexities introduced by combinations of abiotic and biotic factors in the field. To better understand the ecological relevance of deoxygenation events on coral reef biology, it is critical to compare responses to deoxygenation in both controlled experiments and in the field.

**FIGURE 1:**
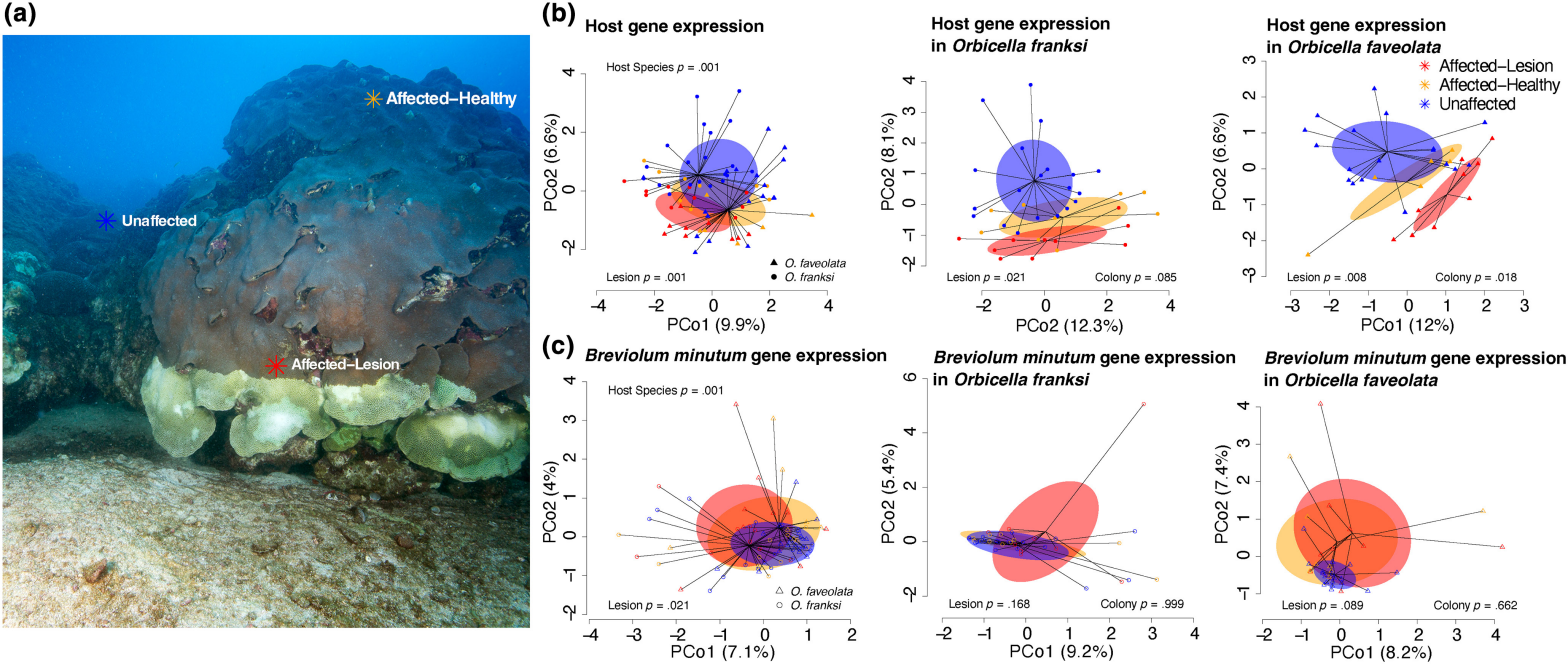
In situ lesions induce changes in host and symbiont gene expression. Sampling strategy during the localized mortality event, photo credit NOAA/G.P. Schmahl (a). Principal coordinate analysis of coral host (b) and symbiont (*Breviolum minutum*) (c) gene expression in affected and unaffected colonies of *Orbicella faveolata* or *Orbicella franksi* after a localized mortality event at East Flower Garden Bank. Colors denote if the sample was collected adjacent to lesions (AL, affected‐lesion), on a healthy region of an affected colony (AH, affected‐healthy), or on a completely unaffected colony (U, unaffected). *p*‐values generated by permutational multivariate analysis of variance using distance matrices indicate significant effects of host species and lesion status on coral host and symbiont gene expression.

In July 2016, a localized mass mortality event (LME) was observed at East Flower Garden Bank (EFGB) of FGB National Marine Sanctuary. This event was speculated to be caused by terrestrial freshwater runoff combined with upwelling that created a low oxygen layer surrounding areas of high coral cover on the EFGB (Kealoha et al., 2020). Deoxygenation events on coral reefs are typically observed in near‐shore coral reef habitats (Nelson & Altieri, 2019), where there is a higher frequency of localized nutrient inputs, making this offshore event surprising. An alternative explanation is that EFGB was inundated with dense, high salinity brine originating from the underlying salt dome after displacement of rock associated with fault activity (Bright & Gittings, 2023). The FGB exists atop salt domes at the edge of the continental shelf approximately 200 km from the Texas–Louisiana border, which is outside of the low oxygen dead zone associated with the Mississippi River delta (Bianchi et al., 2010). The EFGB in particular sits over a fault zone (Bright & Gittings, 2023). During this LME event, low visibility water was observed along with high invertebrate mortality on the benthos of the reef cap (20 m), with white microbial mats extending along coral colonies and sponges (Johnston et al., 2019). At some surveyed sites on the EFGB, up to 80% of reef‐building corals experienced partial or full mortality (Johnston et al., 2019), whereas no mortality was observed at the West FGB (WFGB), despite being only 20 km apart. In surveys 1 week following the event, there was no direct evidence of either hypoxic conditions or brine, thus whatever conditions caused the localized mortality had since dissipated (Bright & Gittings, 2023; Johnston et al., 2019). Here, we assay gene expression responses of two congeneric coral species (*Orbicella faveolata* and *Orbicella franksi*) and their intracellular symbionts (*Breviolum minutum*) at the affected EFGB, and the unaffected WFGB to assay if the mortality lesions observed coincided with a response to deoxygenation. Tissue samples were collected and whole‐genome gene expression profiling was performed on both unaffected colonies and affected colonies (i.e., those observed with recent tissue mortality). For affected colonies, tissues were sampled from two locations: (1) a healthy area of the affected colony, and (2) a paired sample on the same individual immediately adjacent to the lesion edge (Figure 1a). To assess whether the molecular responses of these corals were consistent with deoxygenation stress, a controlled laboratory experiment was performed on *O. faveolata* fragments collected from the EFGB, and whole‐genome gene expression profiling was also performed on these samples following 12 h of low oxygen exposure. Together, these experiments offer a unique opportunity to use organismal molecular responses to reveal the possible complex axis of stress in the environment, assess ecologically relevant core molecular responses to low oxygen, and to assess the potential differences between complex environmental conditions experienced in the field relative to the controlled conditions of the lab.

## 2 METHODS

### 2.1 Field observations and sampling

Tissue fragments were collected in situ via SCUBA from *O. faveolata* and *O. franksi* coral colonies from East (*n* = 58) and West (*n* = 18) FGB from August 5 to 7, 2016, shortly after the first documentation of the LME (July 25, 2016; permit number FGBNMS‐2014‐001). All coral samples were collected between the hours of 12:00 and 16:00 h during peak sunlight hours and between 21 and 27 m depth. Affected corals were only observed on EFGB. Two tissue samples were collected from affected colonies: tissue adjacent to the apparent lesion (AL, *N* = 19; *n* = 9 *O. faveolata*, *n* = 10 *O. franksi*) and apparently healthy tissue ahead of the lesion (AH, *N* = 19; *n* = 9 *O. faveolata; n* = 10 *O. franksi*) (Table S1). Healthy tissue was collected from 38 additional colonies that showed no visible signs of stress from the EFGB (U, *N* = 20; *n* = 10 *O. faveolata; n* = 10 *O. franksi*) and WFGB (U, *N* = 18; *n* = 8 *O. faveolata; n* = 10 *O. franksi*). A microchisel and hammer were used to remove ~1 cm^2^ of coral tissue. Once tissue samples were removed from colonies, they were immediately preserved at depth in pre‐labeled upside‐down 15 mL Falcon tubes containing 200 proof molecular grade EtOH free of air bubbles. Once samples were at the surface, they were transferred into 1.5 mL tubes with fresh 200 proof molecular grade EtOH, and were maintained as cold as possible until arrival at the University of North Carolina at Chapel Hill where they were moved to long‐term storage at −80°C. All sample information including location of collection, species, and colony status/location on colony are in Table S1.

### 2.2 Ex situ deoxygenation challenge experiment

Fragments (~10 cm × 10 cm) from eight *O. faveolata* colonies were collected from 21 to 23 m depth at the EFGB under permit FGBNMS‐2018‐006 on August 2–3, 2018, aboard the R/V Pelican. These fragments were maintained in flow‐through seawater overnight on deck, after which they were maintained in flow‐through ambient conditions on land overnight at Moody Gardens Aquarium. Colonies were then wrapped in wet paper towels and dry shipped to Boston University's Marine Invertebrate Research Facility (MIRF). Upon arrival at MIRF, colonies were acclimated for several weeks after which they were cut into equal sized fragments using a diamond bandsaw (Gryphon AquaSaw). Fragments of three unique genotypes of sufficient size (~4–9 cm^2^ in area) were used in this experiment. Fragments were returned to a holding tank and maintained at ambient oxygen conditions (28°C, 35 ppt salinity and approximately 7 mg O_2_ L^−1^) on a 12:12 h day:night light cycle (50 μmol photons m^−2^ s^−1^) for 62 days of recovery, during which they were fed freshly hatched brine shrimp twice weekly. The purpose of this recovery period was to eliminate the stress response from handling and shipment. However, the recovery period also likely influenced gene expression patterns relative to reef‐type expression, and also resulted in a shift of symbiont community (Figure S1e). However, because all fragments were maintained in the same aquaria conditions, any differences in baseline gene expression should be controlled for in our comparisons between experimental conditions.

Following recovery, individual fragments (*N* = 12; four fragments from each of the three genets) were placed in airtight jars (one fragment per jar) containing approximately 200 mL of artificial seawater at 28°C and 35 ppt salinity. Six jars (two per genet) were deoxygenated by bubbling N_2_ gas through a small hole in the lid of the jar until oxygen levels measured below 1 mg O_2_ L^−1^ using an oxygen logger (FireSting). The average O_2_ levels at the start of the experiment in normoxic control jars was 6.9 ± 0.08 mg O_2_ L^−1^ and the average O_2_ levels in anoxic treatment jars was 0.083 ± 0.017 mg O_2_ L^−1^. Corals were not fed during the anoxia 12‐h challenge experiment. All jars were placed in an environmental chamber maintained at 28°C in complete darkness for 12 h, which spanned the normal 12‐h dark period to avoid disrupting circadian rhythms. Oxygen levels in each jar were re‐measured again at the end of the 12‐h challenge experiment. Average O_2_ levels in normoxic control jars after the 12‐h period was 6.1 ± 0.51 mg O_2_ L^−1^ and average O_2_ levels in anoxic treatment jars was 0.58 ± 0.21 mg O_2_ L^−1^. All coral fragments appeared healthy, with no visual signs of excess mucus production or loss of tissue integrity. Corals were photographed with a Google Pixel2 using a Coral Health Chart for size and color standard. Two‐dimensional surface area of each coral fragment was estimated using the “Freehand Tool” in ImageJ. Tissue was immediately preserved in cold 200 proof molecular grade ethanol and stored at −80°C prior to gene expression library preparation.

### 2.3 Whole‐genome gene expression library preparation

RNA was isolated from preserved coral tissue samples from both in situ and ex situ samples using the RNAqueous Total RNA Isolation Kit (Invitrogen) according to manufacturer's recommendations. Coral tissue was removed from the skeleton using disposable blades and was then centrifuged to remove any remaining skeletal debris. After elution, RNA was DNased using 10 × DNase I (Thermo Fisher Scientific). First‐strand cDNA synthesis from a target of 1 μg DNased RNA per sample, cDNA amplification, barcoding, and sample pooling were performed according to protocols previously described (Lohman et al., 2016; Wright et al., 2019). A total of 76 libraries were prepared from samples collected from the in situ LME and 12 libraries were prepared from samples in the deoxygenation challenge experiment (Table S1). Libraries from the in situ samples were sequenced on the HiSeq 2500 (Illumina) at the University of North Carolina (Chapel Hill, NC) and libraries from the deoxygenation challenge experiment samples were sequenced on the HiSeq 2500 (Illumina) at Tufts University Core Facility (Boston, MA). Sequenced reads have been uploaded to the National Center for Biotechnology Information Short Read Archive (SRA) under accession number PRJNA994102.

### 2.4 Gene expression analyses

Raw reads were trimmed of excess adaptors and low quality reads were discarded using FASTX toolkit (Hannon, 2009). To determine dominant algal symbiont genera hosted by both field‐collected corals from the LME and corals included in the deoxygenation experiment, we mapped trimmed TagSeq reads to a combined symbiont reference composed of transcriptomes from four Symbiodiniaceae genera: *Symbiodinium* (formerly Clade A), *Breviolum* (formerly Clade B), *Cladocopium* (formerly Clade C), and *Durusdinium* (formerly Clade D) (Bayer et al., 2012; Ladner et al., 2012) using Bowtie2 (Langmead & Salzberg, 2012). For field collected samples from the localized mortality event (Field LME), samples hosted predominantly symbionts in the genus *Breviolum* (Figure S1), and thus filtered reads were re‐mapped to concatenated scaffolds of the *O. faveolata* genome and *B. minutum* transcriptome (Parkinson et al., 2016; Prada et al., 2016) using Bowtie2 (Langmead & Salzberg, 2012). While *Orbicella* spp. sampled at the FGB have been found to exclusively host *B. minutum* (Green et al., 2014), corals can modulate their algal communities depending on environmental conditions (Buddemeier & Fautin, 1993; Cunning et al., 2015; Grottoli et al., 2014; Jones et al., 2008). Indeed, the ex situ colonies of *O. faveolata* that had been conditioned in aquaria and used in the deoxygenation experiment were found to host predominantly *Durisdinium* (Figure S1), and thus ex situ deoxygenation samples were re‐mapped to concatenated scaffolds of the *O. faveolata* (Prada et al., 2016) and *Durusdinium trenchii* (Shoguchi et al., 2021) genomes using Bowtie2 (Langmead & Salzberg, 2012). Gene counts were compiled using htseq‐count (Putri et al., 2022) for coral genomes and custom perl scripts for algal symbionts. All custom perl scripts can be found at https://github.com/z0on/tag‐based_RNAseq.

The remaining statistical analyses were performed in R (v 4.2.1). For both gene expression datasets, genes with mean counts less than 3 across all samples were removed and outlier samples were identified using *arrayQualityMetrics* (Kauffmann et al., 2009). Outlier samples that failed two out of three outlier criteria (distance between arrays, array intensity distribution, and individual array quality) were removed from subsequent analyses. Genes mapping to host and symbiont were subset into two separate dataframes, which were analyzed separately. For the field LME dataset, Wald tests were performed in DESeq2 v.1.38.1 (Love et al., 2014), examining contrasts between species (*O. faveolata* vs. *O. franksi*), bank (WFGB vs. EFGB), and all lesions status combinations (healthy region of affected colony [AH], adjacent to lesion on affected colony [AL], and unaffected colony [U]; full model: design = ~species + lesion + bank). Adjusted *p*‐values were calculated using the Benjamini–Hochberg method (Benjamini & Hochberg, 1995). To further examine species‐specific responses to the LME, the coral dataset was subset by species and Wald tests were run to identify contrasts between banks and lesion status. For the ex situ deoxygenation experiment, Wald tests were performed contrasting treatment (control vs. deoxygenation) and genet ID (1, 2, 3; full model: design = ~treatment + genet).

To determine functional enrichment in response to LME and ex situ deoxygenation challenge, Gene Ontology (GO) enrichment analyses were performed for each contrast for hosts and algal symbionts in each dataset using sign‐adjusted *p*‐values with the GO‐MWU package (Wright et al., 2015). To assess divergence in overall gene expression responses between samples, permutational analysis of variance was performed using the *Adonis* function implemented in the *vegan* package v.2.6‐4 (Dixon, 2003) (field LME: design = ~species + bank + lesion; ex situ deoxygenation: design = ~treatment + genet).

To determine if there were conserved responses between the field LME dataset and ex situ deoxygenation, a KOG functional analysis using the KOG‐MWU package (Dixon et al., 2015) was performed using log_2_ fold change (LFC) values for contrasts between lesion status in the field LME dataset and treatment (control vs. deoxygenation) in the ex situ deoxygenation dataset. Contrasts within the field LME dataset included comparisons between all three lesion statuses: (1) AL versus AH, representing differences between tissue adjacent to lesions and tissue from the same colony on healthy tissue far from a lesion; (2) AH versus U, representing differences between healthy tissue on affected colonies and unaffected colonies; and (3) AL versus U, representing differences between tissue adjacent to lesions and unaffected colonies. Additionally, to assess conservation of responses across other published coral environmental stress datasets, KOG comparisons were additionally performed on the following datasets: *O. faveolata* adults affected with stony coral tissue loss disease (SCTLD) compared to unaffected colonies (Traylor‐Knowles et al., 2021); adult deoxygenation exposure versus control conditions in *Acropora tenuis* and *Acropora selago* (Alderdice et al., 2021); the “Type A” general coral stress response in *Acropora millepora* (Dixon et al., 2020); response to white syndrome lesions in *Acropora hyacinthus* (Wright et al., 2015); and *Orbicella* spp. responses to freshwater runoff associated with hurricane Harvey at the FGB (Wright et al., 2019). These comparisons reveal potential functional correlations between coral responses from our dataset and others, which may give insight into common molecular responses across similar stressors and the potential role of low oxygen on the localized mortality event.

## 3 RESULTS

### 3.1 The localized mortality event induced coral host gene expression changes indicative of host metabolic programming

The localized mortality event (LME) led to visible lesions on both *O. franksi* and *O. faveolata* colonies at East Flower Garden Bank (EFGB), but not at West Flower Garden Bank (WFGB; Figure 1a). Tag‐seq profiling resulted in an average of 7.1 million raw reads per sample (±221,834 SEM) and an average of 806,524 reads per sample (±27,533 SEM) after trimming, deduplication, and quality filtering (Table S2). It is important to note that in Tagseq data processing, PCR‐duplicates are removed so each read represents a true transcript unlike other forms of RNAseq, so this depth of coverage is sufficient to explore gene expression patterns (Lohman et al., 2016). For field localized mortality event (field LME) samples, mapping of reads from both species to the combined *O. faveolata* and *B. minutum* genomes resulted in an average 92.8% ± 0.22 SEM mapping efficiency (Table S2) representing 11,365 host genes and 12,714 symbiont genes with a base mean >3. Of this filtered data, four samples in the host dataset and three samples in the symbiont dataset were identified as outliers and removed from subsequent analyses (Table S2).

Coral hosts exhibited 1415 differentially expressed genes (*p*_adj_ < .05; DEGs) between species (*O. faveolata* vs. *O. franksi*; 4.34% of genes mapped) and 41 DEGs between banks (EFGB vs. WFGB; 0.13% of genes mapped). Lesion status (PERMANOVA *p* = .001, *F* = 1.75) explained 4.4% of the variation in host gene expression (Figure 1b). Coral host DEGs associated with adjacency to lesions were also identified (Table S4): comparisons between samples adjacent to lesions (AL) and healthy samples on affected colonies (AH) exhibited 181 DEGs (0.56% of genes mapped) while comparisons between AL and unaffected colonies (U) exhibited 499 DEGs (1.53% of genes mapped). There were zero DEGs between AH and U samples.

Symbiont gene expression showed similar responses as their coral hosts however, they exhibited a smaller overall magnitude of transcriptomic responses (Table S5). Host species ID (PERMANOVA *p* = .001, *F* = 1.67) and lesion status (PERMANOVA *p* = .021, *F* = 1.18) explained the most variation in symbiont gene expression, with no significant effect of bank (PERMANOVA *p* = .062, *F* = 1.18) (Figure 1). Symbionts exhibited 49 DEGs between host species (*O. faveolata* vs. *O. franksi*; 0.13% of genes mapped) and 21 DEGs between banks (EFGB vs. WFGB; 0.05% of genes mapped). Wald test comparisons between AL and AH samples exhibited 26 DEGs (0.07% of genes mapped) while comparisons between AL and U samples exhibited 36 DEGs (0.09% of genes mapped). Similarly to host gene expression, zero DEGs between AH and U samples were observed for the algal symbiont. DEGs between lesion comparisons in *B. minutum* were almost exclusively unannotated, with no representative GO terms, while DEGs between host species include a 60S ribosomal protein, a component of a potassium voltage‐gated channel, Methyltransferase FkbM, and DEAD‐box ATP‐dependent RNA helicase 21, among others (Table S5). Furthermore, *B. minutum* GO terms enriched in the comparison between host species include RNA processing (GO:0006396, *p* = .0065), lipid metabolic process (GO:0006629; GO:0044255, *p* = .01), and cell motility (GO:0048870, *p* = .03) (Table S9).

GO enrichment analyses of gene expression patterns observed during the LME event showcased that coral host tissue adjacent to lesions (AL) differentially regulated mitochondrial processes (Table S8). GO categories including oxidative phosphorylation (GO:0006119; *p*_adj_ = .04), mitochondrial protein complex (GO:0098798, *p*_adj_ = .0002), and mitochondrial respirasome (GO:0005746; GO:0070469; GO:0098803; *p*_adj_ = .01) were enriched in comparisons between samples ahead of the lesion (AL) and healthy tissue on affected colonies (AH) (Table S8). KOG analysis provides further evidence of differential expression of mitochondrial processes associated with adjacency to lesions through differential regulation of energy production and conversion (*p*_adj_ = .0058) (Table S11). Adjacency to lesions was also associated with differential regulation of signal transduction mechanisms as evidenced through KOG enrichment (*p*_adj_ = .00087) and GO enrichment of proton transmembrane transporter activity (GO:0015078; *p*_adj_ = .03).

### 3.2 Ex situ deoxygenation leads to downregulation of cytoskeletal rearrangement and upregulation of stress response mechanisms

Average raw reads per sample for the ex situ deoxygenation experiment were 11.6 million (±1,056,326 SEM) and an average of 2.4 million (±208,808 SEM) reads per sample were maintained after trimming, deduplication, and quality filtering (Table S3). Mapping of reads to the combined *O. faveolata* and *D. trenchii* genomes resulted in an average 86.1% (±0.0079 SEM) mapping efficiency (Table S3) representing 14,176 host genes and 16,222 symbiont genes with a base mean >3. After filtering, one sample was identified in both host and symbiont datasets as an outlier and was removed from subsequent analyses (Table S3). While removal of this sample reduced the sample size of one genet in one treatment below a standard of 3, we maintain the remainder of the analysis on treatment combined across all genets.

**FIGURE 2:**
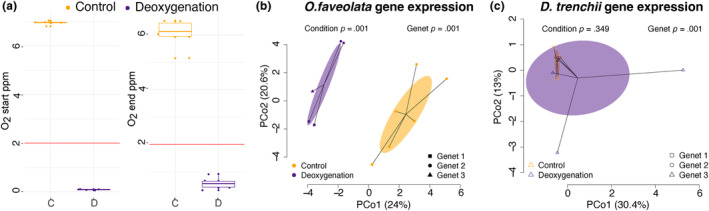
Controlled deoxygenation experiment reveals differences in coral host and symbiont gene expression. Oxygen concentrations at the start and end of the experiment in the control (C) and deoxygenation (D) conditions. The red line indicates hypoxic conditions (a). Principal coordinate analysis of coral host (b) and symbiont (*Durusdinium trenchii*) (c) gene expression in three genets of *Orbicella faveolata* exposed to control or deoxygenation conditions, indicated by different colors. *p*‐values generated by permutational multivariate analysis of variance (PERMANOVA) using distance matrices indicate significant effects of condition (deoxygenation vs. control) and genet ID on coral host and symbiont gene expression.

Genet ID (PERMANOVA *p* = .001, *F* = 3.2) and oxygen treatment (PERMANOVA *p* = .001, *F* = 3.9) explained 36.9% and 22.7% of the variation in host gene expression, respectively (Figure 2b). Coral hosts exhibited 1907 DEGs between control and deoxygenation treatments (5.85% of genes mapped) (Table S6). Symbiont gene expression also showed significant effects of genet ID (PERMANOVA *p* = .001, *F* = 1.2), but non‐significant effects of oxygen treatment (PERMANOVA *p* = .35, *F* = 1.0). Furthermore, zero genes were differentially expressed in symbionts under deoxygenation conditions (Table S7). Coral host gene expression responses to deoxygenation were enriched for key stress response mechanisms, including the GO categories of innate immune response (GO:0045087; *p*_adj_ = .004), defense response (GO:0006952; *p*_adj_ = .0003) and DNA repair (GO:0006281; *p*_adj_ = .03) (Table S10). Coral hosts also exhibited enrichment of GO terms associated with cytoskeletal rearrangement, including cilium movement (GO:0003341; *p*_adj_ = .01), microtubule bundle formation (GO:0001578; GO:0035082; *p*_adj_ = .02), and significant KOG enrichment of cytoskeleton (*p*_adj_ = .0023) (Table S12).

### 3.3 Minimal evidence for a core molecular response to deoxygenation

**FIGURE 3:**
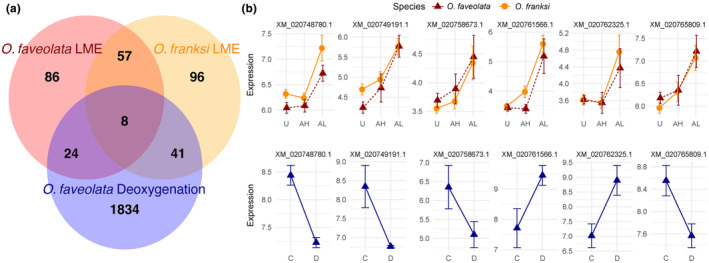
Limited overlap of differentially expressed genes between *Orbicella* spp. during the localized mortality event (LME) and *Orbicella faveolata* under ex situ deoxygenation conditions. (a) Venn diagram indicating the overlap of differentially expressed genes (DEGs, *p*_adj_ < .05) between *O. faveolata* and *Orbicella franksi* responses during the LME, and a controlled deoxygenation experiment on *O. faveolata*. (b) Gene expression patterns of the 6/8 overlapping DEGs in both datasets with putative annotations. XM_020748780.1—DMGDH, a putative dimethylglycine dehydrogenase; XM_020749191.1—prostatic spermine‐binding protein‐like; XM_020758673.1—galaxin‐like; XM_020761566.1—glutamic acid‐rich protein‐like; XM_020762325.1—GP2‐like, a pancreatic secretory granule membrane major glycoprotein; XM_020765809.1—calumenin‐like. Lesion statuses are categorized as unaffected (U), healthy region of affected colony (AH), and adjacent to lesion on affected colony (AL). Ex situ conditions are categorized as control (C) or deoxygenation (D).

To explore species‐specific responses to the LME, the gene expression dataset was subset by species and genes significant (*p*_adj_ < .05) for either AL comparison (AL and AH, AL and U) were combined as the overall LME response. When comparing the LME response between these two species, 65 genes were similarly differentially expressed in response to AL status (Figure 3). These genes include betaine–homocysteine *S*‐methyltransferase (BHMT), peroxiredoxin‐6 (PRDX6), cation channel sperm associated auxiliary subunit gamma (CATSPERG), and cartilage oligomeric matrix protein (COMP), among other mostly unannotated genes (Table S4).

Next, we compared these species‐specific responses to the LME with the ex situ deoxygenation experiment, which revealed eight commonly differentially expressed genes across species and experiments (Figure 3), including putative proteins dimethylglycine dehydrogenase, prostatic spermine‐binding like protein, galaxin‐like, a glutamic acid‐rich protein, calumenin‐like, pancreatic secretory granule membrane major glycoprotein GP2‐like and two unannotated genes (XM_020751503.1 and XM_020752970.1) (Figure 3b). Of these eight common DEGs, six were annotated, and four of the genes were expressed in opposite directions between the LME and ex situ deoxygenation experiments (Figure 3b). It should be noted that the LME samples with lower sequencing depth overall likely resulted in fewer highly expressed genes (11,365 host genes represented) for this analysis than the ex situ deoxygenation samples (14,176 host genes represented). Thus, minimal overlap in similarly expressed genes could be reflective of differences in sequencing depth and coverage. We also examined if the LME and the deoxygenation experiment induced expression of hypoxia inducing factor (HIF) genes, which have been shown to be key activators regulating the multitude of different hypoxia response pathways in environmentally sensitive *A. selago* (Alderdice et al., 2021). We find that both *O. faveolata* and *O. franksi* upregulated HIF1A in response to the LME, although this result was not significant and thus was excluded using a *p*_adj_ cutoff of <.05 (*p*_adj_ = .085; Figure S2). HIF1A was also significantly upregulated under ex situ deoxygenation conditions (*p*_adj_ = 3.9e‐12; Figure S2). HIF1AN and EGLN1, both HIF suppressors, were not represented in the LME dataset, potentially due to low read counts. HIF1AN was not significantly differentially expressed in the deoxygenation dataset (*p*_adj_ = .3), while EGLN1 was similarly not represented.

**FIGURE 4:**
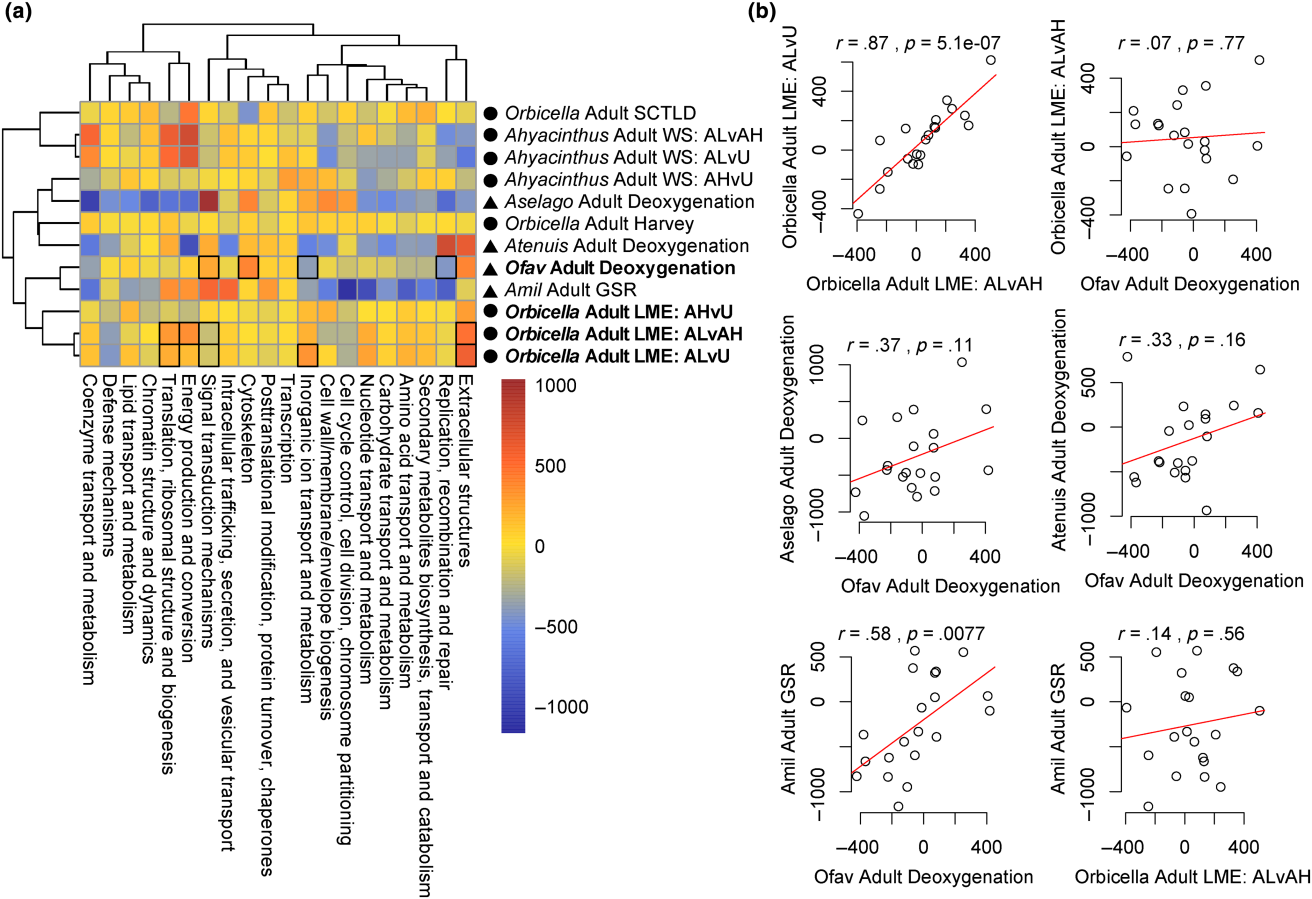
Correlations between functional gene expression categories among the localized mortality event and ex situ deoxygenation experiments and other published stress response datasets. (a) KOG correlation heatmap of gene expression datasets generated in this manuscript (in bold), and previously published related stress responses datasets: *Orbicella faveolata* adults affected with stony coral tissue loss disease (SCTLD) lesions (Traylor‐Knowles et al., 2021); adult deoxygenation exposure in *Acropora tenuis* and *Acropora selago* (Alderdice et al., 2021); *Orbicella* spp. adults after hurricane stress (Wright et al., 2019); *Acropora hyacinthus* affected with white syndrome (Wright et al., 2015); and the coral general stress response (GSR) in *Acropora millepora* (Dixon et al., 2020). Bolded squares indicate KOG category significance after a Mann–Whitney *U* test at *p*_adj_ < .05. Color scale indicates KOG delta rank values. Circles indicate datasets collected in the field, while triangles indicate datasets generated from controlled laboratory experiments. (b) Correlations of KOG delta rank values between datasets. Detailed model information for each comparison can be found in Table S11. It should be noted that gene annotations may vary between species, although KOG annotations were assigned through the same database.

To better understand differences in broader functional enrichment between these datasets and other coral stress response datasets, we used a KOG clustering analysis. Similar to comparative patterns between the LME and ex situ deoxygenation datasets, correlations between datasets using delta ranks of KOG terms revealed that the gene expression responses associated with adjacency to lesions (LME response) were not strongly correlated with the ex situ deoxygenation gene expression response (Figure 4; *r* = .07; *p* = .77). In fact, the LME response was not significantly correlated with exposure to any other stressors observed in both lab and field experiments (Figure 4; Table S11). In contrast, the *O. faveolata* ex situ deoxygenation experiment showed a slight but non‐significant correlation to deoxygenation in *A. selago* (*R^2^* = .37; *p* = .11) and *A. tenuis* (*R^2^* = .33; *p* = .16) (Figure 4) and a significant positive correlation with the generalized stress response in *A. millepora* (Figure 4; *R^2^* = .58; *p* = .0077). The LME response was however not significantly correlated to the generalized stress response in *A. millepora* (Figure 4; *R^2^* = .14; *p* = .56).

## 4 DISCUSSION

### 4.1 Gene expression responses to lesions indicate downregulation of mitochondrial function and upregulation of antioxidants

The localized mortality event (LME) observed at Flower Garden Banks (FGB) is hypothesized to have been driven by inundation of dense anoxic brine (Bright & Gittings, 2023), or a combination of freshwater surface inputs, mostly derived from the Mississippi/Atchafalaya and Texas rivers, combined with upwelling (Kealoha et al., 2020). Freshwater combined with upwelling could have stratified waters within the EFGB where reef‐building coral resides, leading to photosynthesis inhibition and increased respiration, likely depleting oxygen concentrations, ultimately creating acute, severe low oxygen conditions for a period of days (Kealoha et al., 2020). Here, we sampled affected coral colonies of *O. faveolata* and *O. franksi* adjacent to lesions, on healthy regions of the same colonies, and on apparently healthy colonies at both the WFGB and EFGB to reveal molecular signatures of host and symbiont responses to this in situ LME. We found that coral host tissue adjacent to lesions differentially regulated mitochondrial processes such as oxidative phosphorylation, cellular respiration, transporter activity, and signal transduction. These data indicate host metabolic reprogramming, which could reflect multiple downstream pathways activated by Hypoxia Inducible Factor 1 Subunit Alpha (HIF1A) expression (Nelson & Altieri, 2019; Vaquer‐Sunyer & Duarte, 2008). While we observed an increase in HIF1A expression in coral tissue samples adjacent to mortality regions, these expression differences were not significantly different from healthy samples, so it is unclear if HIF1A activated these potential downstream pathways, despite downstream evidence of their activation.

One of the pathways activated by HIF1A is a switch from aerobic to anaerobic metabolic pathways (Alderdice et al., 2021, 2022; Murphy & Richmond, 2016; Nelson & Altieri, 2019; Vaquer‐Sunyer & Duarte, 2008). We observed some evidence for this shift, with comparisons between ahead of the lesion (AL) and healthy (AH) LME samples showing differential regulation of oxidative phosphorylation and proton transmembrane transport (Table S8). During periods of low oxygen, the tricarboxylic acid cycle (TCA) cycle and oxidative phosphorylation are inhibited as aerobic respiration shifts to anaerobic respiration (Wooldridge, 2013). Deoxygenation experienced at night further triggers calcification mechanisms, which operate through transmembrane protein pumping of H^+^ and Ca^2+^ ions between calcifying fluid and the coelenteron (Wooldridge, 2013). Therefore, our results suggest shifts in metabolic function near the lesion site, highlighting the importance of oxygen for physiological processes such as respiration, photosynthesis and calcification (Deleja et al., 2022; Wooldridge, 2013).

Modulation in mitochondrial functioning also leads to production of reactive oxygen species (ROS), which, in excess, can contribute to symbiosis breakdown (Downs et al., 2002; Lesser et al., 1990; Smith et al., 2005) and is associated with DNA damage induced by low oxygen conditions (Deleja et al., 2022). We observe upregulation of antioxidant peroxiredoxin‐6‐like (PRDX6) in coral hosts adjacent to lesions. PRDX6 is a key detoxifying enzyme involved in protection from oxidative stress, suggesting host mitigation of increased ROS. PRDX6 also plays a role in immune defense (Knoops et al., 2016), where it is upregulated in mice macrophages during infection, potentially to protect immune cells from oxidative damage (Diet et al., 2007). Thus, upregulation of PRDX6 could indicate a role in the response to the environment, innate immunity from opportunistic invading pathogens, or both. PRDX6 has been implicated in multiple responses to the environment, including associations with SCTLD transmission in *O. faveolata* (Traylor‐Knowles et al., 2021) and heat stress in *A. millepora* (Petrou et al., 2021). However, differential regulation of antioxidants is more generally observed in multiple stress responses including coral disease (Traylor‐Knowles et al., 2021; Wright et al., 2015, 2017), salinity stress (Aguilar et al., 2019), heat stress (Bellantuono et al., 2012; Dixon et al., 2015; Mayfield et al., 2018), and acidification (Davies et al., 2016). Similar patterns of gene expression responses including modulation of mitochondrial processes and antioxidants were also found in *Orbicella* spp. at the FGB following hurricane Harvey (Wright et al., 2019), suggesting similar stress response mechanisms between the FGB LME and response to hurricane Harvey conditions.

Betaine‐homocysteine *S*‐methyltransferase 1‐like (BHMT) is upregulated in coral hosts adjacent to lesions in both species. BHMT is the first step in the catabolism of glycine betaine (GB), a common osmolyte that is scavenged by coral hosts and catabolized to serine (Ngugi et al., 2020), with the second step in the pathway mediated by dimethylglycine dehydrogenase (DMGDH). Both BHMT and DMGDH are upregulated in coral hosts adjacent to lesions, suggesting a host response to a potential excess release of GB associated with the LME. BHMT was also found to be upregulated in white‐plague disease‐infected *O. faveolata* (MacKnight et al., 2022). Taken together, these observations suggest a potential undescribed role of GB catabolism in response to coral disease and this in situ LME, perhaps indicating its involvement in the coral environmental stress response (ESR) more generally, in addition to its observed role in coral‐Symbiodiniaceae symbiosis (Ngugi et al., 2020).

### 4.2 Species‐specific responses to the localized mortality event

While the functional responses of both *Orbicella* species to the localized mortality event (LME) were similar, there were also unique gene expression responses between the two species (Figure 3). The LME had a stronger effect on cell growth pathways, including down regulating genes involved in the regulation of extent of cell growth (GO:0061387; GO:0030516), in *O. faveolata* compared to *O. franksi*. In contrast, *O. franksi* downregulated genes associated with chromosome condensation (GO:0000793) and DNA metabolic processes (GO:0006259). We also observed differential regulation of HORMAD1 in *O. faveolata* but not in *O. franksi*, which is an evolutionarily conserved gene involved in regulating meiotic checkpoints through detection of asynapsis during prophase (Kogo et al., 2012). Overall gene expression patterns in both species are characteristic of a coral environmental stress response (ESR) (Barshis et al., 2012; Dixon et al., 2015), but unique DEGs in each species suggest they likely employed species‐specific responses to the LME or that the magnitude of response was divergent in some gene pathways. Similar species‐specific responses to deoxygenation have been described in sister *Acropora* species as well (Alderdice et al., 2021). Specifically, *A. selago* showed evidence of bleaching susceptibility and elevated expression of HIF1A under deoxygenation compared to controls while *A. tenuis* did not (Alderdice et al., 2021). Here we ran a KOG correlation analysis to identify if functional responses of these two *Acropora* species were significantly correlated with *Orbicella* spp. responses to the LME in our study (Figure 4). We found that overall functional responses to the LME were unique from functional responses of two *Acropora* deoxygenation responses, despite activation of some similar molecular pathways, which may reflect either taxon‐specific responses to deoxygenation, or that coral responses to the LME are different than what is experienced under laboratory deoxygenation conditions. While we did not account for differences in gene annotation methods between different studies and species in our analysis, we used higher level summaries of different functional responses at the KOG level to compare functional responses between datasets, with gene‐level KOG annotations generated through the same database (https://github.com/z0on/emapper_to_GOMWU_KOGMWU?tab=readme‐ov‐file).

### 4.3 Ex situ deoxygenation induced a characteristic environmental stress response

To compare *Orbicella* spp. LME responses with responses to controlled deoxygenation conditions in the lab, we ran an additional ex situ deoxygenation experiment on *O. faveolata* collected from the EFGB (Figure 2a). In these samples, we observed a robust host response to deoxygenation, including upregulation of defense response pathways, the innate immune response, chemotaxis, and responses to lipids (Table S10). We also observed downregulation of replication, recombination, and repair mechanisms, suggesting an arrest of cellular growth. *Orbicella faveolata* exposed to acute deoxygenation upregulated HIF1A relative to control fragments, which was associated with differential regulation of multiple deoxygenation response pathways, including changes in the generalized coral environmental stress responses (ESR) (Alderdice et al., 2021, 2022; Barshis et al., 2012; Dixon et al., 2015, 2020). For example, Tumor necrosis factor receptor associated factor 3 (TRAF3)—a known immune regulator of the transcription factor NF‐κb in cnidarians—was upregulated under deoxygenation; this gene has been broadly implicated in the coral ESR across diverse taxa (Barshis et al., 2012; Cziesielski et al., 2019; Vidal‐Dupiol et al., 2022). NF‐κb1 itself was also upregulated under deoxygenation (Table S6) and this transcription factor has been widely implicated in the maintenance of symbiosis and ESRs of reef‐building corals and symbiotic anemones (Bove et al., 2022; Fuess et al., 2020; Mansfield et al., 2017; Rivera & Davies, 2021). Despite activation of HIF1A and differential expression of some characteristic downstream response pathways, the functional responses of acute deoxygenation in *O. faveolata* did not correlate with those observed in *A. tenuis* or *A. selago* (Figure 4), emphasizing taxon‐specific responses to acute deoxygenation. These differences included a noticeable lack of differential expression of heat shock proteins in *O. faveolata*, with the exception of HSPA4L (Table S6), which have broadly been observed in other taxa (Cziesielski et al., 2019).

*Orbicella faveolata* exposed to our acute deoxygenation treatment exhibited downregulation of genes related to replication, recombination, and repair. This response is similar to that exhibited by *A. selago*, which was sensitive to ex situ deoxygenation, but contrasts the response of *A. tenuis*, which upregulated this functional class of genes (Figure 4; Alderdice et al., 2021). For context, the experiments in *Acropora* spp. were exposed to nighttime oxygen levels around 2 mg O_2_ L^−1^ (Alderdice et al., 2021), slightly higher than our experimental exposures but within the standards of deoxygenation experiments. Both studies had deoxygenation occur during the dark period of a 12:12 dark cycle. Furthermore, *O. faveolata* gene expression was significantly correlated with the generalized coral ESR (Figure 4; Dixon et al., 2020), indicating that, while this species may be physiologically robust to deoxygenation as observed in Bocas del Toro, Panama (Johnson, Swaminathan, et al., 2021), *O. faveolata* from the FGB still mounts a characteristic ESR to acute deoxygenation induced under extreme deoxygenation conditions in the lab.

While we note that we did not control for pH in this experiment, which would have been slightly elevated in the deoxygenation treatment due to the N_2_ addition, we believe it is unlikely that any potential differences in pH are driving the molecular responses we observed. The duration of the exposure was much shorter than typical acidification studies and the oxygen and pH stress were very likely opposing (i.e., deoxygenation treatment likely had higher pH than control treatment and thus had less stressful pH). In addition, other studies have shown that the differences in pH between deoxygenation and ambient treatments are quite small (Pontes et al., 2023) and thus unlikely to have a significant impact on our results.

### 4.4 Muted response of Symbiodiniaceae to LME and ex situ deoxygenation compared to host

*Orbicella* spp. at the FGB are commonly dominated by algal symbionts in the genus *Breviolum* (Green et al., 2014). Mapping of our LME samples to multiple symbiont genera references confirmed that these colonies hosted *Breviolum*. However, *O. faveolata* genets sourced from the FGB and maintained in aquaria prior to the ex situ deoxygenation experiment were found to host a high proportion of reads mapping to *Durusdinium* (Figure S1). While this result is surprising given the symbiont fidelity observed in the field, shifts in dominant symbiont type under different environmental conditions are not uncommon in corals (Buddemeier & Fautin, 1993; Cunning et al., 2015; Grottoli et al., 2014; Jones et al., 2008). Coral host gene expression is also known to be modulated by the symbiont type hosted across a diversity of coral species (Barfield et al., 2018; Cunning & Baker, 2020; Desalvo et al., 2010; Helmkampf et al., 2019; Strader & Quigley, 2022), thus gene expression differences we observed between LME and ex situ deoxygenation samples could reflect host responses to homologous and heterologous symbioses, respectively (Bove et al., 2022). Due to the confounding overlap between sample type (LME or ex situ deoxygenation) and dominant symbiont (*Breviolum* or *Durisdinium*), we are unable to tease apart symbiont specific effects on the LME or ex situ deoxygenation. Thus, differences observed between these datasets, and the lack of overlap in their overall response to deoxygenation conditions, could reflect hosting homologous versus heterologous symbiont types. Future experiments should therefore consider investigating the possible interactive effects of symbiosis on the host response to deoxygenation.

Symbiont gene expression responses in both datasets were minimal compared to those of their hosts, with an order of magnitude fewer differentially expressed genes (DEGs) in the LME samples and zero DEGs in the ex situ deoxygenation samples (Figures 1c and 2c). Muted algal responses to stress are common in gene expression experiments (Aichelman et al., 2024; Barshis et al., 2014; Baumgarten et al., 2013; Davies et al., 2018; Leggat et al., 2011) and may suggest that the alga's residence inside the host buffers them from external stress. Despite a muted molecular response of Symbiodinaceae compared to the host (Figure 1), we still found significant differentially expressed genes in *B. minutum* when compared between host species, site, and affected‐lesion samples (AL vs. AH and AL vs. U comparisons) (Table S5). When comparing gene expression responses between host species, we observe differences in lipid metabolism, RNA processing, and cellular movement. Many lipids are synthesized in symbiont cells in hospite and are transferred to the host (Crossland et al., 1980; Maor‐Landaw et al., 2020; Peng et al., 2011), therefore differential expression observed here may represent adaptations leading to host species‐symbiont specificity. While our data cannot resolve whether or not these two species host distinctly adapted *B. minutum* genotypes or strains, this would be an interesting avenue for future research.

### 4.5 *Orbicella* gene expression during LME likely reflects a complex response to multiple factors, not deoxygenation in isolation

Multiple lines of evidence suggest that *Orbicella* spp. sampled after the LME did not elicit a characteristic response to deoxygenation: (1) Neither species showed significant upregulation of HIF1A (Figure S2), (2) *Orbicella* spp. gene expression responses to the LME did not significantly correlate with functional molecular responses to deoxygenation from other studies, to other abiotic stressors, to the generalized coral stress response, or even to other field‐collected samples in response to different stressors (Figure 4), and (3) In comparisons between LME and ex situ deoxygenation samples, we only found eight genes that were commonly expressed between datasets (Figure 3), none of which were components of what has previously been described as the hypoxia induced response (Alderdice et al., 2021, 2022), although these results may be confounded by different dominant symbionts between the datasets. Consequently, we do not observe *Orbicella* spp. in the LME field samples differentially regulating many of the core pathways in the HIF activated hypoxia response system, including shifts in differential regulation of heat shock proteins (Alderdice et al., 2021, 2022), suggesting species‐specific responses to deoxygenation or that the LME event was a more complex stressor than those involved in experimental studies. Of the eight DEGs commonly shared between both species and datasets, two were upregulated in both species in response to the LME and in the ex situ deoxygenation experiment: glutamic acid‐rich protein‐like and GP2‐like (Figure 3). GP2 is a putative pattern recognition glycoprotein previously implicated in Chomera and Symbiodiniaceae establishment in *A. millepora* (Mohamed et al., 2018, 2020), thus highlighting an interesting possible association between deoxygenation and the regulation of symbiosis.

Differences in stress‐response timing, as well as multiple other biotic and abiotic differences between field and lab environments, likely drive transcriptomic differences observed between the LME and ex situ deoxygenation, as well as between our results and other published transcriptomic responses to deoxygenation challenge in corals. Field conditions experienced by the LME corals were likely a complex combination of factors that include, but are not limited to, deoxygenation. During the LME, EFGB experienced more upwelling of deeper waters than WFGB, which coincides with reduced pH, higher DIC, higher ammonium, higher salinity, and shifts to deep‐water microbial communities (Doyle et al., 2022; Kealoha et al., 2020). In addition, strong shifts in the local biotic community were observed, which may have also contributed to responses (Johnston et al., 2019). While corals in the field may have been exposed to critically low oxygen levels similar to our laboratory manipulations (Johnston et al., 2019; Le Hénaff et al., 2019), we did not sample tissue until mass mortality had already been observed in the field. In contrast, we sampled asymptomatic tissue after 12‐h of acute deoxygenation stress in the lab‐based experiment. Thus, differences in responses observed between the ex situ deoxygenation challenge here and in other studies may also be explained by differences in the duration and magnitude of deoxygenation challenge, combined with the myriad of additional abiotic stressors associated with the LME.

Our lack of a characteristic deoxygenation response across previous experiments may also be explained by evidence that *O. faveolata* is more robust to deoxygenation than more sensitive species such as *Acropora cervicornis* (Johnson, Swaminathan, et al., 2021). Notably, this LME elicited tissue mortality denounced by distinct lines (Figure 1a); therefore, we suspect that unique and disparate microenvironments likely resulted in stressful conditions that led to tissue loss. Our data suggest that these stressful microenvironments may have included deoxygenation, but more likely reflect a complex combination of abiotic factors that generated a stress response, potentially involving dense brine (Bright & Gittings, 2023). Because dense brine is often associated with low oxygen, it is possible that low oxygen conditions contributed to the LME associated lesions; however, our data show that tissue loss here was not strictly due to deoxygenation. Regardless, both *Orbicella* spp. were able to maintain tissue survival despite significant tissue loss on portions of the colony, indicative of either the notable robustness of *Orbicella* spp. to stressors, or the severity of differences in distinct microenvironments experienced throughout the colony. Additionally, LME samples collected here were sampled after tissue mortality had occurred; therefore, samples were taken from polyps that survived but were adjacent to tissue mortality, representing what may have been a latent regeneration response. However, prior studies show that acute deoxygenation stress does reduce oxygen consumption in *O. faveolata* but does not induce shifts to anaerobic respiration (Gravinese et al., 2022), similar to the response we observed at the gene expression level. This could have repercussions for the larger Caribbean, which will continue to experience an increasing number of deoxygenation events (Pezner et al., 2023). While *O. faveolata* shows high susceptibility to white syndrome disease (MacKnight et al., 2022), and similar differential regulation of the extracellular matrix and immune response as observed in our study, it appears to be less susceptible to SCTLD than many other Caribbean species (Aeby et al., 2019; Traylor‐Knowles et al., 2021), and generally robust to deoxygenation stress (this study; Johnson, Swaminathan, et al., 2021). These results highlight the importance of comparing in situ gene expression responses with controlled lab experiments, because responses of corals in the field are not restricted to differences in a single factor in isolation, as they often are in lab experiments.

### 4.6 Contributions of this study to conservation efforts

Here, we profile molecular responses of *Orbicella* spp. to a localized mortality event potentially associated with a dense brine release and low oxygen conditions (Bright & Gittings, 2023). These molecular responses indicate some evidence of stress, likely in response to distinct microenvironments that occurred throughout different regions of the colonies. Despite these sometimes distinct microenvironments, likely involving some level of deoxygenation, regions adjacent to tissue mortality exhibited some level of resilience. These results emphasize the importance of studying and conserving populations with high coral cover to maintain genetic diversity within the Gulf of Mexico and Caribbean. The FGB has potential to act as a source population to degraded reefs in the Caribbean, through facilitating the dispersal of propagules from relatively healthy colonies with high fecundity (Davies et al., 2017; Rippe et al., 2017), therefore should be an area of high conservation priority in the Anthropocene.

## AUTHOR CONTRIBUTIONS

**Marie E. Strader:** Formal analysis (equal); project administration (equal); visualization (equal); writing – original draft (equal); writing – review and editing (equal). **Rachel M. Wright:** Conceptualization (equal); data curation (equal); formal analysis (equal); investigation (equal); methodology (equal); project administration (equal); visualization (equal); writing – review and editing (equal). **Ariel K. Pezner:** Writing – review and editing (supporting). **Marissa F. Nuttall:** Data curation (equal); investigation (equal); writing – review and editing (equal). **Hannah E. Aichleman:** Data curation (equal); methodology (equal); writing – review and editing (equal). **Sarah W. Davies:** Conceptualization (equal); data curation (equal); funding acquisition (equal); investigation (equal); project administration (equal); supervision (equal); writing – review and editing (equal).

## Supporting information


Figures S1–S2

Tables S1–S12

## Data Availability

The datasets generated for this study can be found in the National Center for Biotechnology Information Short Read Archive (SRA) under accession number PRJNA994102. Analysis scripts available at: http://github.com/mariestrader/FGB_LME.
