# Supplementary material for: Intersection of coral molecular responses to a localized mortality event and ex situ deoxygenation
Source: Ecol Evol. 2024 Apr 23;14(4):e11275. doi: 10.1002/ece3.11275 (PMC11036075; doi:10.1002/ece3.11275)
Supplement: Supplementary file 1 — Figures S1–S2 [file ECE3-14-e11275-s001.docx]

**Intersection of coral molecular responses to an in situ localized mortality event and ex situ deoxygenation**

Marie E. Strader_,_ Rachel M. Wright, Ariel K. Pezner, Marissa F. Nuttall, Hannah E. Aichleman, Sarah W. Davies

Supplementary Figures


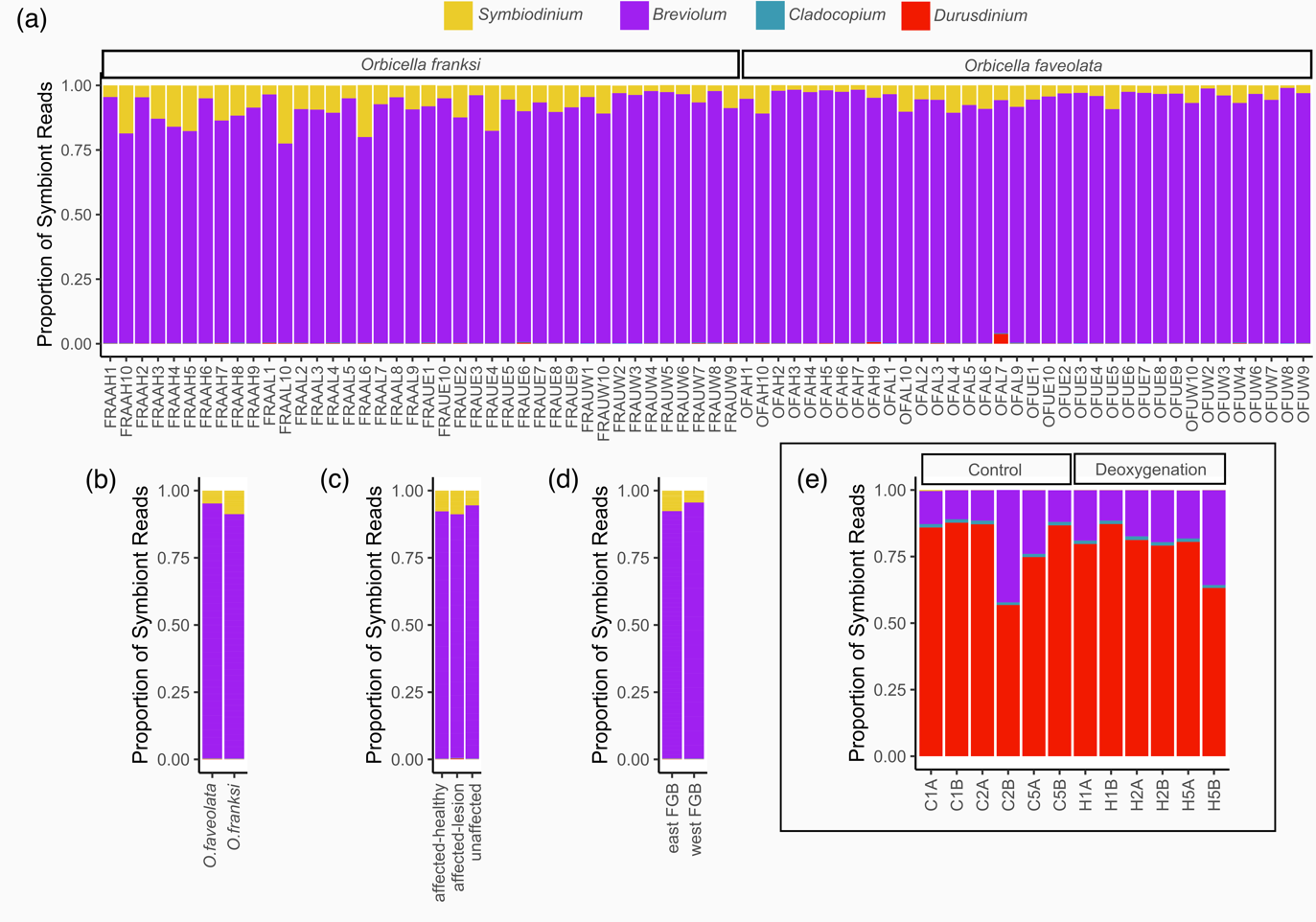


## ***Supplementary Figure 1: Dominant Symbiodiniaceae genus differs between field LME and ex situ deoxygenation samples.*** (a) Proportion of RNAseq reads mapping to each of 4 Symbiodiniaceae transcriptomes for all LME samples, (b) overall proportions by species, (c) colony tissue location, and (d) collection location (EFGB or WFGB). Inset (e) shows proportion of reads mapping to each of the four Symbiodiniaceae genera in the *Orbicella faveolata* ex situ deoxygenation samples.

***Supplementary Figure 2: HIF1A expression.*** (a) HIF1A mean normalized expression across sample types (U, unaffected; AH, affected-healthy; AL, affected-lesion) for both species sampled from the LME. (b) HIF1A mean normalized expression in control (C) and deoxygenation (D) treatments from the ex situ deoxygenation experiment.
