# Supplementary material for: Intersection of coral molecular responses to a localized mortality event and ex situ deoxygenation
Source: Ecol Evol. 2024 Apr 23;14(4):e11275. doi: 10.1002/ece3.11275 (PMC11036075; doi:10.1002/ece3.11275)
Supplement: Supplementary file 2 — Tables S1–S12 [file ECE3-14-e11275-s002.zip › TablesS1-S12_Legends.docx]

Table S1: Sample sizes for both in situ and ex situ experiments.

Table S2: Sequencing details for in situ field samples including total raw reads, total trimmed reads, mapping efficiencies and outlier results for each sample.

Table S3: Sequencing details for ex situ samples including total raw reads, total trimmed reads, mapping efficiencies and outlier results for each sample.

Table S4: Host Differentially Expressed Genes with significance (padj<0.05) in comparisons between colony status (affected lesion (AL), affected healthy (AH), unaffected (U)), bank (east (E), west (W)), species (*Orbicella franksi* (FR), *Orbicella faveolata* (OF))

Table S5: *Breviolum* Differentially Expressed Genes with significance (padj<0.05) in comparisons between colony status (affected lesion (AL), affected healthy (AH), unaffected (U)), bank (east (E), west (W)), species (*Orbicella franksi* (FR), *Orbicella faveolata* (OF))

Table S6: Host Differentially Expressed Genes with significance (padj<0.05) in comparisons between hypoxia and control in lab experiment with FGB *Orbicella faveolata*

Table S7: *Durusdinium* Differentially Expressed Genes with significance (padj<0.05) in comparisons between hypoxia and control in lab experiment with FGB *Orbicella faveolata*

Table S8: Host GO terms with significant (padj<0.05) enrichment in comparisons between colony status (affected lesion (AL), affected healthy (AH), unaffected (U)), bank (east (E), west (W)), species (*Orbicella franksi* (FR), *Orbicella faveolata* (OF))

Table S9: Symbiont GO terms with significant (padj<0.05) enrichment in comparisons between colony status (affected lesion (AL), affected healthy (AH), unaffected (U)), bank (east (E), west (W)), species (*Orbicella franksi* (FR), *Orbicella faveolata* (OF))

Table S10: GO terms with significant (padj<0.05) enrichment in comparisons between 3 unique genets of *Orbicella faveolata* exposed to control and hypoxia conditions under lab conditions

Table S11: KOG results with field samples from the FGB localized mortality event (LME). Comparisons are between colony status (affected lesion (AL), affected healthy (AH), unaffected (U))

Table S12: KOG results from lab hypoxia experiment with *Orbicella faveolata* from the FGB
